# GCAC: galaxy workflow system for predictive model building for virtual screening

**DOI:** 10.1186/s12859-018-2492-8

**Published:** 2019-02-04

**Authors:** Deepak R. Bharti, Anmol J. Hemrom, Andrew M. Lynn

**Affiliations:** School of Computational and Integrative Sciences, Jawaharlal Nehru University, New Delhi, 110067 India

**Keywords:** Predictive model building, Reproducible results, Galaxy workflow system, High throughput screening, Drug discovery, R statistical package, Cheminformatics

## Abstract

**Background:**

Traditional drug discovery approaches are time-consuming, tedious and expensive. Identifying a potential drug-like molecule using high throughput screening (HTS) with high confidence is always a challenging task in drug discovery and cheminformatics. A small percentage of molecules that pass the clinical trial phases receives FDA approval. This whole process takes 10–12 years and millions of dollar of investment. The inconsistency in HTS is also a challenge for reproducible results. Reproducible research in computational research is highly desirable as a measure to evaluate scientific claims and published findings. This paper describes the development and availability of a knowledge based predictive model building system using the R Statistical Computing Environment and its ensured reproducibility using Galaxy workflow system.

**Results:**

We describe a web-enabled data mining analysis pipeline which employs reproducible research approaches to confront the issue of availability of tools in high throughput virtual screening. The pipeline, named as “Galaxy for Compound Activity Classification (GCAC)” includes descriptor calculation, feature selection, model building, and screening to extract potent candidates, by leveraging the combined capabilities of R statistical packages and literate programming tools contained within a workflow system environment with automated configuration.

**Conclusion:**

GCAC can serve as a standard for screening drug candidates using predictive model building under galaxy environment, allowing for easy installation and reproducibility. A demo site of the tool is available at http://ccbb.jnu.ac.in/gcac

**Electronic supplementary material:**

The online version of this article (10.1186/s12859-018-2492-8) contains supplementary material, which is available to authorized users.

## Background

Over the past few decades, the time and cost of drug development have increased. Today, it typically takes about 10–15 years and costs up to $1300 - $1500 million to convert a promising new compound into a drug in the market, which reflects the complexity of the drug discovery process [1]. One challenge for the scientific community is to bring down cost and time for drug development. The computational studies of biological and chemical molecules for drug-like properties falls under a separate branch of science called Cheminformatics. It includes high-throughput screening of chemical molecules, which is useful to screen large chemical library using knowledge-based rules to narrow down chemical space for identifying promising drug-like molecules with certain physico-chemical properties. In Cheminformatics, two major computational screening approaches are available in an early phase of drug discovery. First, the Structure-based Virtual Screening (VS) and second, Ligand-based VS [2]. The structure based VS includes high-throughput docking of candidate molecules to target receptors to rank them based on their predicted binding affinity [3]. This approach is relatively fast compared to conventional methods such as whole cell bioassay and in-vivo screening of individual candidates. However, it is not as accurate due to a multilevel preparation of ligands and insufficient information about the local and global environment for efficient binding prediction besides being time consuming when the compound library is large [4]. Studies reveal that ligand-based VS methods have the higher potency of hits identified than the structure-based VS [5, 6]. The Ligand-based VS includes 2D, 3D similarity search, pharmacophore mapping and Quantitative Structure Activity Relationship (QSAR) modelling. The 2D similarity based methods outperform 3D similarity search methods. However, the accuracy of search results heavily relies on a number of available positive cases because the fundamental idea of ligand-based VS is to correlate structure similarity to functional similarity. In the present study, we focus on Ligand-based VS method, especially on QSAR based modelling, and describe the development of an installable platform containing all the steps required for predictive model building and screening using a web-interface deployed using the Galaxy Workflow system.

### Predictive model building in drug discovery process

Ligand-based VS is an example of empirical research where prediction is made for the new case, based on the observed pattern in data under study. The empirical vHTS include predictive model building in which different Machine Learning (ML) methods are combined with data mining to extract hidden patterns and important physical factors for drug-likeness. Predictive model building is a widely used term in the field of economics and has been used in cheminformatics for vHTS of drug-like molecules for various diseases [7–10]. There are several standalone packages and executables available for many ML methods to perform data mining and predictive model building such as Weka [11], RapidMiner [12] and KNIME [13] but their applications in bioinformatics and cheminformatics are not comprehensive, leaving the scope for alternatives. None of the above mentioned tools provides a completely reproducible platform for descriptor calculation, model building, prediction tasks as well as user-friendly appearance.

**Fig. 1:**
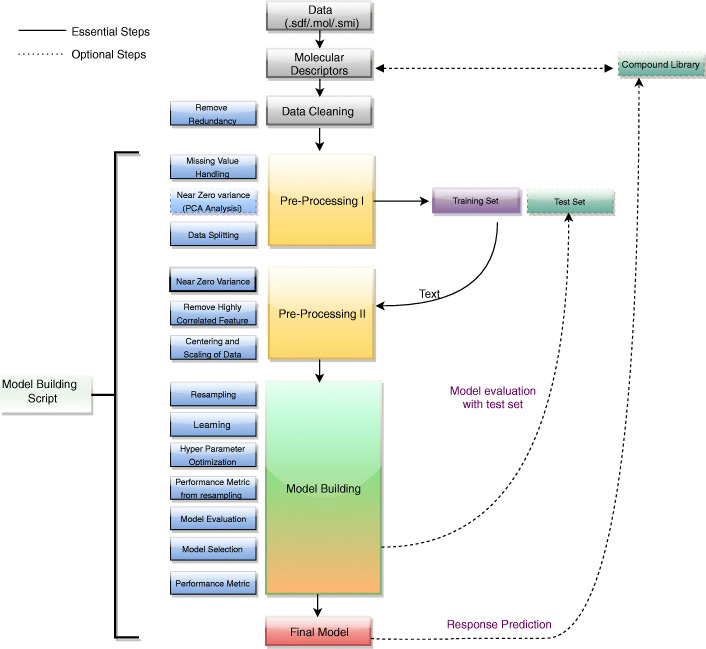
QSAR based predictive model building - a typical protocol used in GCAC: The initial data is molecular structural information file (SDF/MOL/SMILE) which can be used to generate molecular descriptors. Once descriptors are generated, data cleaning is performed which ensures the removal of data redundancy. Preprocessing is performed in two steps - first, the removal of missing values and near zero variance features as they are not useful for model building. The input data is split into training and test datasets. The training data set is used for model building and test data is used for model evaluation. In a second step of preprocessing, further treatment is applied as per selected method for model building. It includes removal of zero variance features, highly correlated values, centering and scaling of data in the training data. In the model building step, learning and hyper-parameter optimization is facilitated using resampling, internal cross-validation and performance evaluation over the set of parameters chosen. The most accurate model is selected and evaluated on test data set. The selected model is saved and utilized further when a new set of compounds need to be predicted from a set of compounds of unknown activity

QSAR model based VS uses the power of ML and data mining for accurate bioactivity prediction [14]. Lack of a web-enabled reproducible QSAR model based prediction platform also causes a serious impediment to empirical Virtual High Throughput Screen (vHTS). In the field of drug discovery, a reproducible screening workflow is indeed essential due to the high cost of the procedure [15]. Cloud-based virtual machines with a pre-set computing environment provides a solution to overcome this problem. However, web-enabled QSAR modelling and prediction is still under development with only a few successful implementations e.g. CADDSuite [16], but an absence of many widely accepted classifiers for model building and lack of information about response value (e.g., binding energy, IC 50 value) restricts its advantages. Another web-enabled QSAR modelling tools is ChemModLab [17] which provides many utilities such as descriptor calculation, model building and prediction on unlabelled set but it lacks the generation of dynamic reports for model building and various cross-validation methods to ensure robustness of the model. Although ChemModLab is an excellent and sophisticated implementation of the computational drug discovery process, its usage is limited due to lack of availability for locally installable versions. Therefore, a robust web-enabled, as well as locally installable platform, is strongly required to expedite large molecule library screen and model building ensuring reproducibility of results. An ideal protocol for model building applied to QSAR is shown in Fig. 1, used to design the system described in this manuscript.

### Galaxy workflow system

**Fig. 2:**
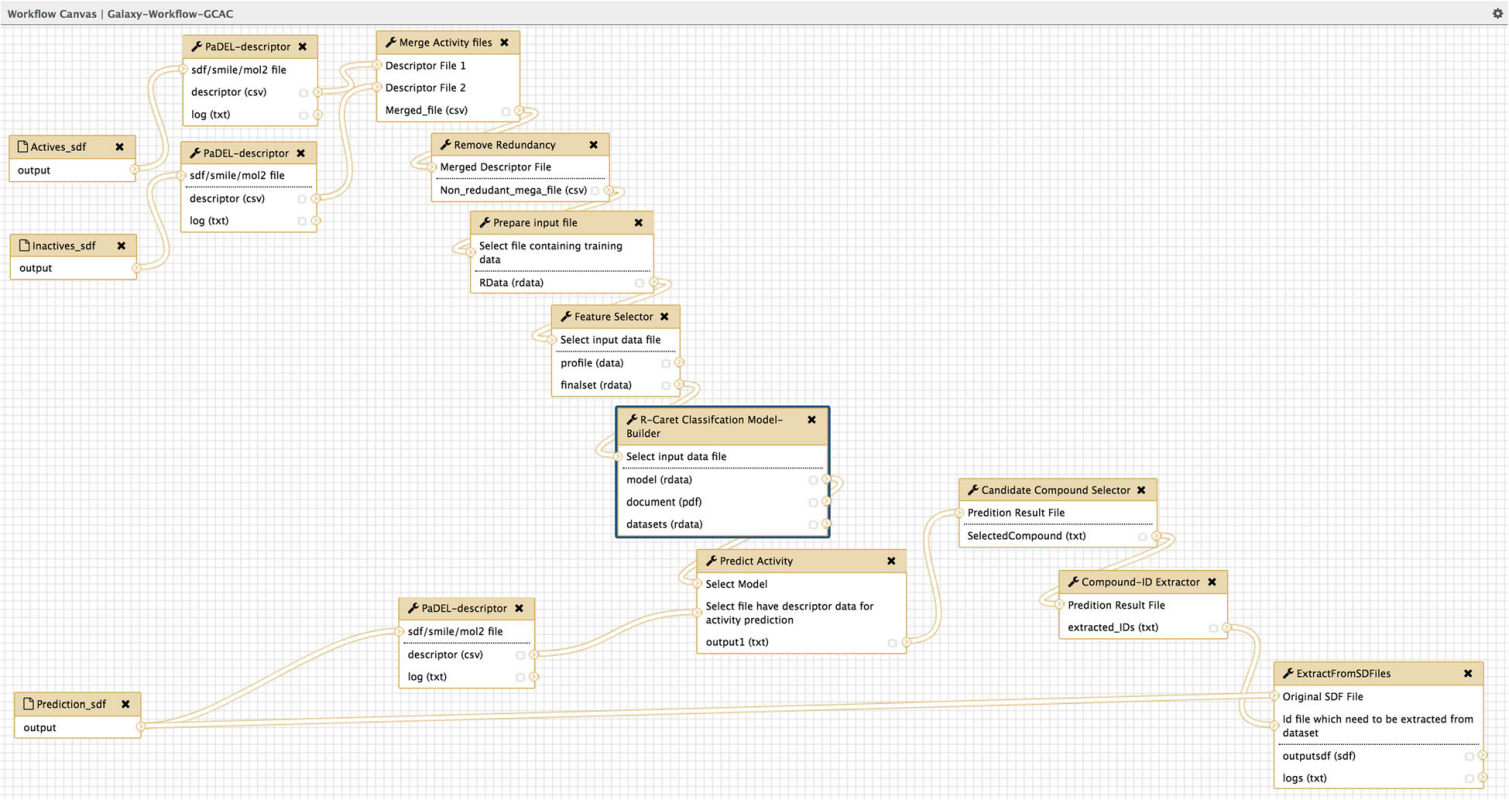
GCAC example workflow. The figure is a screenshot of the galaxy workflow canvas showing the arrangements of individual elements which can be used to create a workflow for model building and prediction of active molecules. Each element is described in more detail in Table 1 of this manuscript

Galaxy is an open source workflow system that can subsume almost all command line based bioinformatics tools. In computational biological analysis, the issue of transparency, reproducibility and accessibility can be addressed by using Galaxy [18]. It has a large community supportand features like history, workflow, data library and pages leveraged for sharing of analysis pipelines among users. Galaxy can be easily integrated with cluster and cloud computing, which is the biggest requirement for continuously growing biological data and multilevel analysis. We have extended the work by incorporating the R Sweave script with open source web-based Galaxy framework as a part of model building tool to foster “reproducible research”. The Galaxy workflow for predictive model building can be easily understood by Fig. 2, which shows the tools developed as part of GCAC linked together to form a workflow. The availability of the script as a Galaxy tool extend its usefulness. The Galaxy framework provides a graphical interface to users and facilitates creating reusable computing pipelines for execution, analysis and publishing of results. Notwithstanding the applicability of Galaxy for virtual screening and preclinical analysis, only a few tools have been developed in Galaxy.

## Implementation

Here we introduce a galaxy suite of tools collectively referred to as GCAC (Galaxy-enabled- Compound Activity Classification), which allows the use of predictive analysis within the Galaxy platform. We used Python, R statistical package [19], Sweave [20] and bash to develop wrappers for already existing open-source packages as well as in-house scripts for various tasks (Table 1).

**Table 1:** List of Galaxy Tools developed as part of GCAC: The GCAC suite comprises mainly four major tasks. Each task contains one repository and at least one tool associated with it. The GCAC tools are available in galaxy main toolshed (https://toolshed.g2.bx.psu.edu/repository?repository_id=351af44ceb587e54)

Major Tasks	Toolshed Repositories	Tool Name	Description
Descriptor Calculation	padel_descriptor_calculation	PaDEL­Descriptor	calculates descriptors for active and inactive datasets.
	activity_files_merge	Merge Activity Files	assigns response values and merges positive and negative datasets.
	redundant_entries_remove	Remove Redundancy	removes redundant entries of molecules.
Feature Selection	feature_selection	Feature Selector	selects best features subset
Model Building and Prediction	csv_to_rdata	Prepare input file	converts csv_files to RData format
	rcaret_classification_model	R-Caret Classification Model-Builder	builds classification model using ‘caret’ R package
	activity_predict	Predict Activity	predicts activity of molecules using their descriptor file (prediction set) and supplied model.
Candidate Compound Extraction	candidate_compound_select	Candidate Compound Selector	selects compound name or ids of interesting molecules based on certain cutoff range.
	compound_id_extract	Compound­ID Extractor	extracts compound IDs to be used in downstream compound extraction from sdf files
	mayatools_extract	ExtractFromSDFiles	provides sdf file of extracted compounds from the prediction set

We developed a wrapper for the PaDEL-descriptor package for descriptor calculation and R-caret [21] package for predictive model building. The power of caret package is embedded within sweave template scripts for generation of dynamic reports. Sweave allows statistical analysis and documentation simultaneously which is reproducible over any similar or identical computing environment. For extraction of compounds from rest of molecules which qualifies in prediction set, we developed a MayaChemTools [22] based wrapper. To choose the optimal set of features we developed the caret based feature selection tool. There are many additional intermediate helper tools designed to connect these major tools. The GCAC tools are hosted on galaxy main tool shed (https://toolshed.g2.bx.psu.edu/repository?repository_id=351af44ceb587e54) as well as a virtual machine (http://ccbb.jnu.ac.in/gcac). Providing an open source resource for QSAR predictive analysis will undoubtedly improve accessibility, transparency and reproducibility in in-silico drug discovery process.

## Results and discussion

### Data

**Fig. 3:**
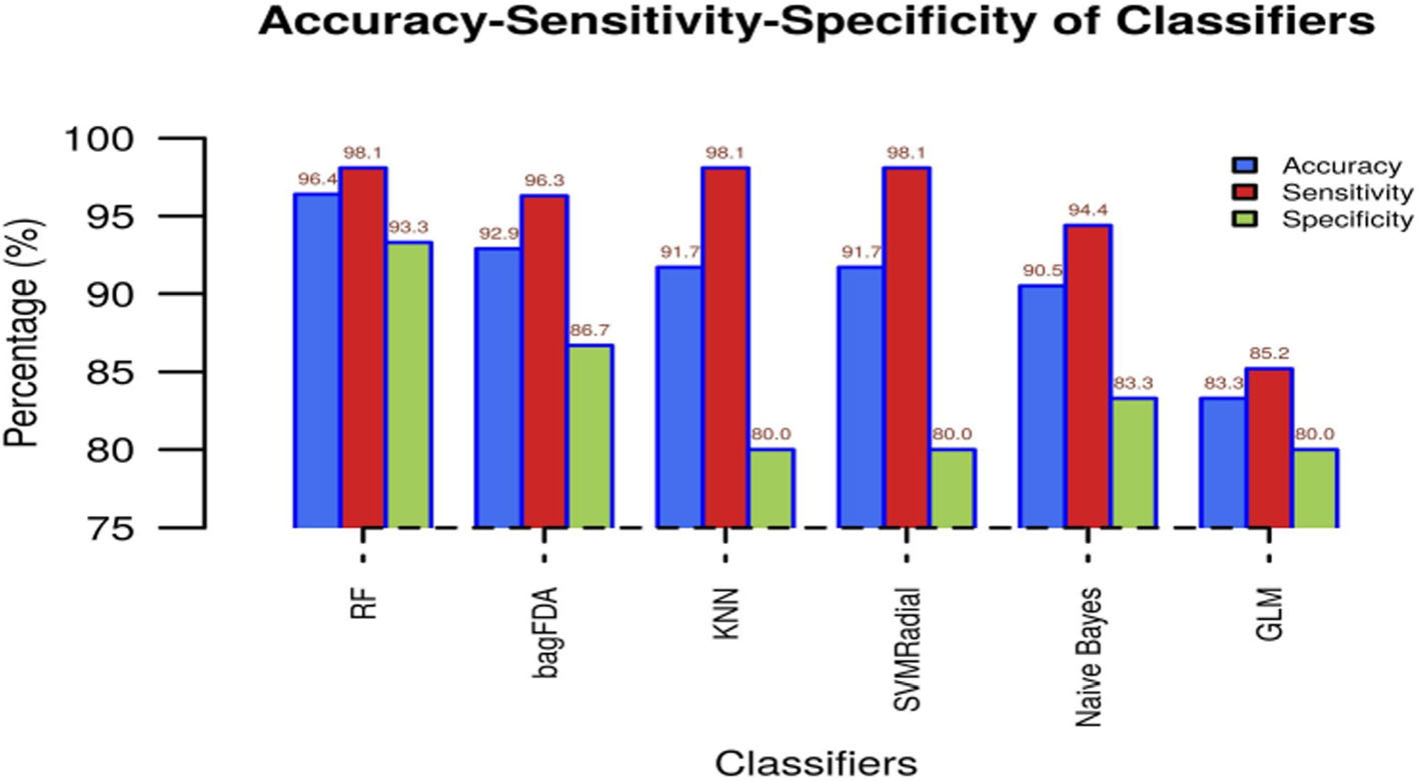
Performance Metrics for Fontaine Dataset. The performance metrics for the ‘*fontaine dataset*’ a standard dataset described in more detail in the Data section of this manuscript, used to validate the protocol with some example models. RF - Random Forest, bagFDA - bagging with Flexible Discriminant Analysis, KNN - K- Nearest Neighbours, SVMRadial - Support Vector Machine using a Radial kernel function, NaiveBayes - Naive Bayes, GLM - Generalised Linear Model

The initial data for predictive model building can be any file in sdf, smile or mol format. There are a multiple sources of data present in publically available chemical databases like PubChem [23], ZINC [24], ChEMBL [25], etc. The PubChem bioassays are the biggest resource of chemical data. ZINC contains commercially-available compounds for virtual screening while ChEMBL is a manually curated database of drug-like molecules. All three databases provide chemical data in a various format including sdf, mol, and smile which are required format for initial data used in proposed Galaxy workflow. For demonstration purpose, we selected the standard “Fontaine” data sets [26] for evaluating the performance of proposed galaxy pipeline. The dataset comprises of hepatitis c virus (HCV) NS3 protease inhibitors and acetylcholinesterase (AChE) inhibitors. The HCV NS3 protease is a highly studied serine protease that plays a crucial role in viral replication and well-known drug target [27]. While AChE datasets contain inhibitors of acetylcholinesterase, their activity may result in a rise in acetylcholine concentration in Alzheimer patients [28]. There are total 435 compounds are present in the dataset of which 279 are actives, and 155 are inactive. The protocol performs adequately, as shown in Fig. 3. GCAC offers the user multiple methods for model-building, some of which are superior to earlier published methods, while showing comparable results when the same method is used. Additional file 1: Tables S1 and S2 show details of multiple methods applied to the Fontaine dataset.

### GCAC tool repositories

The GCAC tools are organized into three main directories within one Git Repository: descriptor_calculation, model_building_and_prediction and candidate_compound_extraction. Each of them comprises of one or more subdirectories containing a tool for the particular job. The underlying idea of creating directories is a separation of primary tasks and associated tools - namely 1) descriptor calculation 2) feature selection and model building 3) screening to extract potent candidates.

### Descriptor calculation

In recent years, descriptor based model building are encouraged for faster virtual screening. Many commercial and open source descriptor calculation software like CDK, JOELib, Dragon, and PowerMV [29–32], etc., are available for the user community. PaDEL is open source software for descriptor calculation [33]. It calculates 1785 various 1D, 2D, and 3D descriptors. Additionally, it also calculates 12 types of chemical fingerprints. The input file can be a smile, mol or sdf and output is CSV file of calculated descriptors. We developed Galaxy wrapper for PaDEL-descriptor consisting it’s all essential parameters. There are two helper tools also designed to concatenate files after adding class information, and for eliminating repeated entries which ultimately returns a merged descriptor file having labels (i.e., Class information).

### Feature selection

The objectives of feature (also known as a predictors, attributes, variables, etc.) selection can be summed into three points. First, for improving the prediction performance of the predictors. Second, providing faster and cost-effective predictors for quick learning, and thirdly, providinga better understanding of the underlying rules that generated the data [34]. Featureselection techniques can be summarized into three categories, depending on their integration with the model building process: filter methods, wrapper methods, and embedded methods [35]. Filter methods are computationally fast and easy to implement, but most filter techniques are univariate which fails to identify any interactions among features. Embedded methods are more computationally efficient than wrapper methods but rely on a specified learning algorithm. Wrapper methods outperform filter methods as they search for the optimal set of features, and are sufficient to classify data at the expense of computational cost. Moreover, wrapper methods have the benefits of identifying dependencies among features and the relationships between the feature subset and model selection. As filter methods are insufficient for the optimal feature set and caret has many classifiers with built-in feature selection, we have developed a feature selection tool, using the Recursive Feature Elimination (RFE), a wrapper method for feature selection provided within caret package. After conversion of csv file into RData one can employ feature selection tool for identifying optimal feature subset for model building step. Currently, a user can choose any of four functions (random forest, linear, treebag, and naive bayes) for model fitting and several options for cross-validation measures.

### Model building

Model building is an important aspect of GCAC pipeline. We focused on ensuring its reproducibility and added a feature of automated dynamic report creation that has not been available in most of the predictive analysis pipeline. The report thus generated is vital in context containing information about the computing environment, data properties, statistical measures and their significance. The merged descriptor file obtained after “Descriptor Calculation” step is converted into required input data format (i.e.,RData) and then may optionally be subjected to the feature selection tool or can be used solely for building a model using the tool, “Build-Classification-Model”. At the backend, it uses an R Sweave template which creates a runtime script having information of applied classification method and various other parameters set by the user. It produces three outputs: Model, Document, and Datasets used (i.e., train and test set).

For classification purpose, GCAC provides 15 machine learning (ML) methods for model building including Generalised Linear Model (GLM), Random Forest(RF), Naive Bayes(NB), K- Nearest- Neighbours (KNN), Support Vector Machine (SVM), C5.0, Adaptive Boosting (AdaBoost), etc. Additional file 1: Table S3 contains a list of methods tested, along with tunable parameters. If an imbalanced dataset is used for modelling, GCAC provides sampling methods like “downsample” and “upsample” to ameliorate class information. GCAC also provides options for selecting resample methods such as CV, LOOCV, repeated CV, Bootstrap 632 and boot for cross-validation study. A model is evaluated over many performance metrics like accuracy, sensitivity, specificity, kappa statistics and ROC curve analysis. The pdf document generated consists of preliminary information and properties of the data under study, the applied pre-processing steps, performance measures, graph(s), table(s), and confusion matrix. A well-formatted PDF generation is one of the major features of GCAC pipeline. Additionally, the user has access to train and test datasets, which are used for model building. The model generated can be utilized to predict the activity of unlabelled compound library and may also be employed for making ensembles of various models to improve the predictive power of data mining applications. The prediction result consists of identifier or molecule names along with probability score of being a positive or negative case. A high value indicates a higher chance of belonging to the particular class. Predicted molecules can be extracted from a prediction set at the later stage.

### Extract potential Lead like compounds

Once prediction result obtained, it is essential to extract potential molecules from prediction set for further analysis. We developed Galaxy wrapper tools for three important tasks: selecting interesting molecules using probability score cut-off, input preparation and extraction of molecules into a distinct file. The required format for the prediction set is structure data file (sdf). Based on prediction score, a user may choose interesting molecules which are extracted from prediction set and written into different sdf file using the “MayaChemTools” based Galaxy tool.

## Conclusions

The cost and time are the greatest bottlenecks in drug discovery process. It’s essential that drug discovery stages remain as replicable, transparent, reviewable and accessible as much as possible. The GCAC platform in Galaxy helps to facilitate all of these goals. In the present study, the PaDEL-descriptor tools can be used to calculate molecular descriptors using publicly available chemical datasets (PubChem, zinc, ChEMBL etc.). The most influencing feature subset can be obtained by using the RFE based feature selection tool. The model building module provides many commonly used state-of-the-art classifiers available in caret package. The workflow uses R-caret - where parameters specific to a model-building method are optimised within the model building process. Though the default model used is PLS, the user may choose from a large range of model-building methods, which is dependent of available computational time and expected accuracy. From our preliminary results on the use of the protocol, different models may perform better with different data sets. To address the problem of large class imbalance in datasets, we implemented downsampling and upsampling methods to optimise ratio of positive and negative cases. Each model can be evaluated using widely accepted performance measures like AuROC, Accuracy, Sensitivity, Specificity and Kappa statistics. The best model selected can be used to predict the activity of unknown compound library and predicted active or positive cases can be extracted using maya tool which may further be subjected to computational analysis.

If the scientific community succeeds to lower the cost and time required for initial drug discovery processes without losing confidence about the reproducibility of results, millions of dollars and many lives will be saved. By applying QSAR based virtual screening, we can reduce the time taken for virtual screening. In silico ADMET test can also be subjected to automation and parallelization using Galaxy workflow system which again will result in lowering time. One of the limiting factors for QSAR based model building is the availability of data for training for “global” model. This problem can be addressed by making “local” models exclusively for given target or chemical-series-specific data.

Future development of GCAC will comprise of three major additions: A wide range of ML methods for classification, open source ADMET tools development and provisioning of target specific models via shared data. As improved and efficient open source packages will be published for descriptor calculation, ADMET prediction and model building, We integrate them accordance to their utility. As more users participate in GCAC user community, sharing of data, tools, and models will eventually bring more attention of the scientific community. The Galaxy workflow system is well adapted for cloud-based resources and which make Galaxy a more reasonable choice for developing pipelines for drug discovery as well as other biological sciences.

## Availability and requirements

Project name: GCAC.

Project home page: https://github.com/LynnLab-JNU/gcac-galaxy-tools

Demo Server: http://ccbb.jnu.ac.in/gcac

Operating system(s): Linux - Developed, tested and distributed as VM with CentOS 7.

Programming language: R, Python, Shell, Bash.

Other requirements: None.

License: MIT License.

Any restrictions to use by non-academics: None.

A list of required dependencies, more information and download links can be found in the documentation available on the demo site at http://ccbb.jnu.ac.in/gcac.

The GCAC module is made available to users via following standard methods.

i. *Provided* via *VirtualBox VM*: - This is the easiest means to get the GCAC module in a standalone VM environment. Users are required to download and import the VM to their VirtualBox environment.
ii. *Provided* via *Toolshed*: - The GCAC module galaxy tools are made available via publicly available Toolshed repository which can be installed via admin interface on running Galaxy server. Users are also required to install system-level dependencies on the Galaxy host machine.

## Additional file

Additional File 1: **Table S1.** Fontaine (Factor Xa) Data set: After feature selection, 201 features remained for model building. Model building was performed on default GCAC-parameters. The bootstrap 632 rule (10 reps) was used for hyper-parameter optimisation. There were 273 active and 151 inactive molecules in complete data set. The model was built using a training set of 340 molecules and evaluated on test set of 84 molecules. **Table S2.** Performance comparison over fontaine data set with previously published results. (In case of multiple modelling conditions, the best result was taken from literature for comparison. All reported work has accuracy reported over training). **Table S3.** List of model-building methods tested and reported in this manuscript, with tunable parameters for each model. (DOCX 23 kb)

## Abbreviations

AchE: Acetylcholinesterase
AdaBoost: Adaptive boosting
CV: Cross-validation
GCAC: Galaxy for compound activity Classification
GLM: Generalised linear model
HCV: Hepatitis c virus
HTS: High throughput screening
KNN: K- Nearest- Neighbour
LBVS: Ligand based virtual screening
LOOCV: Leave one out cross validation
ML: Machine learning
NB: Naive Bayes
QSAR: Quantitative structure activity relationship
RF: Random forest
RFE: Recursive feature elimination
ROC: Receiver operating characteristic
SBVS: Structure based virtual screening
SVM: Support vector machine
