# Supplementary material for: GCAC: galaxy workflow system for predictive model building for virtual screening
Source: BMC Bioinformatics. 2019 Feb 4;19(Suppl 13):550. doi: 10.1186/s12859-018-2492-8 (PMC7394323; doi:10.1186/s12859-018-2492-8)
Supplement: Supplementary file 1 — Table S1. Fontaine (Factor Xa) Data set: After feature selection, 201 features remained for model building. Model building was performed on default GCAC-parameters. The bootstrap 632 rule (10 reps) was used for hyper-parameter optimisation. There were 273 active and 151 inactive molecules in complete data set. The model was built using a training set of 340 molecules and evaluated on test set of 84 molecules. Table S2. Performance comparison over fontaine data set with previously published results. (In case of multiple modelling conditions, the best result was taken from literature for comparison. All reported work has accuracy reported over training). Table S3. List of model-building methods tested and reported in this manuscript, with tunable parameters for each model. (DOCX 23 kb) [file 12859_2018_2492_MOESM1_ESM.docx]

GCAC : Galaxy Workflow System for Predictive Model Building for Virtual Screening

Deepak R. Bharti ^1#^, Anmol J. Hemrom^1#^, Andrew M. Lynn^1∗^

Additional file

**Table S1 : Fontaine (Factor Xa) Data set** : After feature selection, 201 features remained for model building. Model building was performed on default GCAC-parameters. The bootstrap 632 rule (10 reps) was used for hyper-parameter optimisation. There were 273 active and 151 inactive molecules in complete data set. The model was built using a training set of 340 molecules and evaluated on test set of 84 molecules.

| **Method** | **Sensitivity** | | **Specificity** | | **Accuracy** | | **ROC** | | **Kappa** |
| --- | --- | --- | --- | --- | --- | --- | --- | --- | --- |
|  | **Test** | **Train** | **Test** | **Train** | **Overall** | **Train** | **Test** | **Train** |  |
| PLS | 0.981 | 0.969 | 0.867 | 0.951 | 0.94 | 0.96 | 0.976 | 0.986 | 0.867 |
| RF | 0.981 | 0.981 | 0.900 | 0.95 | 0.952 | 0.965 | 0.979 | 0.994 | 0.895 |
| treebag | 0.981 | 0.968 | 0.766 | 0.92 | 0.905 | 0.944 | 0.981 | 0.983 | 0.782 |
| GLM | 0.889 | 0.839 | 0.800 | 0.784 | 0.857 | 0.811 | 0.870 | 0.836 | 0.689 |
| NB | 0.963 | 0.97 | 0.867 | 0.9 | 0.929 | 0.935 | 0.960 | 0.975 | 0.842 |
| SVM-RBF | 0.981 | 0.972 | 0.900 | 0.952 | 0.952 | 0.962 | 0.967 | 0.991 | 0.895 |
| KNN | 0.981 | 0.969 | 0.900 | 0.956 | 0.952 | 0.962 | 0.987 | 0.989 | 0.895 |
| Adaboost | 0.981 | 0.971 | 0.900 | 0.924 | 0.952 | 0.948 | 0.987 | 0.989 | 0.895 |
| Earth | 0.963 | 0.949 | 0.867 | 0.895 | 0.929 | 0.922 | 0.98 | 0.964 | 0.842 |

**Table S2 : Performance comparison over fontaine data set with previously published results.** *(In case of multiple modelling conditions, the best result was taken from literature for comparison. All reported work has accuracy reported over training)*

| Method | Model | Measure | Reported | Obtained | Reference |
| --- | --- | --- | --- | --- | --- |
| CART | treebag | Accuracy | 0.954 | 0.944 | [[1]](https://paperpile.com/c/mCHn6z/M3IY) |
| Random Forest | RF | Accuracy | 0.955 | 0.965 | [[1]](https://paperpile.com/c/mCHn6z/M3IY) |
|  | RF | Accuracy | 0.932  (option1 - Gra)  0.951  (option2 - Fin) | 0.965 | [[2]](https://paperpile.com/c/mCHn6z/UPjC) |
| Naïve Bayes | NB | Accuracy | 0.901  (option1 -Ext)  0.901  (option2 - Fin) | 0.935 | [[2]](https://paperpile.com/c/mCHn6z/UPjC) |
| K -nearest Neighbor | KNN | Accuracy | 0.943 | 0.962 | [[3]](https://paperpile.com/c/mCHn6z/5E2k) |
|  | KNN | Accuracy | 0.93 | 0.962 | [[3], [4]](https://paperpile.com/c/mCHn6z/5E2k+vZOz) |
| Partial least Square | PLS | Accuracy | 0.88 | 0.96 | [[5]](https://paperpile.com/c/mCHn6z/7WtV) |

[1] [D.-S. Cao, Q.-S. Xu, Y.-Z. Liang, X. Chen, and H.-D. Li, “Automatic feature subset selection for decision tree-based ensemble methods in the prediction of bioactivity,” *Chemometrics Intellig. Lab. Syst.*, vol. 103, no. 2, pp. 129–136, 2010.](http://paperpile.com/b/mCHn6z/M3IY)

[2]  [A. A. Rafati-Afshar and A. Bouchachia, “An Empirical Investigation of Virtual Screening,” in *2013 IEEE International Conference on Systems, Man, and Cybernetics*, 2013.](http://paperpile.com/b/mCHn6z/UPjC)

[3]  [F. Buchwald, T. Girschick, M. Seeland, and S. Kramer, “Using Local Models to Improve (Q)SAR Predictivity,” *Mol. Inform.*, vol. 30, no. 2–3, pp. 205–218, Mar. 2011.](http://paperpile.com/b/mCHn6z/5E2k)

[4]  [C. Chu and B. K. Alsberg, “A knowledge-based approach for screening chemical structures within de novo molecular evolution,” *J. Chemom.*, vol. 24, no. 7–8, pp. 399–407, 2010.](http://paperpile.com/b/mCHn6z/vZOz)

[5]  [F. Fontaine, M. Pastor, I. Zamora, and F. Sanz, “Anchor-GRIND: filling the gap between standard 3D QSAR and the GRid-INdependent descriptors,” *J. Med. Chem.*, vol. 48, no. 7, pp. 2687–2694, Apr. 2005.](http://paperpile.com/b/mCHn6z/7WtV)

**Table S3: List of model-building methods tested and reported in this manuscript, with tunable parameters for each model.**

Link to Caret online documentation providing more details. More information at (<https://topepo.github.io/caret/available-models.html> ). For more details please refer the book *Applied Predictive Modelling* by Kuhn and Kjell [6]. The optimal parameters are obtained by predefined grid search.

| **Method /Model** | **type** | **Tunable parameter(s)** |
| --- | --- | --- |
| Partial Least Squares (PLS) | Classification/Regression | ncomp |
| Neural Network (nnet) | Classification/Regression | size,decay |
| Bagged Flexible Discriminant Analysis (bagFDA) | Classification | degree, nprune |
| Multivariate Adaptive Regression Spline (earth) | Classification/Regression | degree, nprune |
| Random Forest (rf) | Classification/Regression | mtry |
| Regularized Random Forest (RRF) | Classification/Regression | mtry, coefReg |
| Support Vector Machines with Linear Kernel (svmLinear) | Classification/Regression | C |
| Support Vector Machines with Polynomial Kernel (svmPoly) | Classification/Regression | degree,scale,C |
| Support Vector Machines with Radial Basis Function Kernel (svmRadial) | Classification/Regression | sigma,C |
| AdaBoost Classification Trees (adaboost) | classification | nIter,method |
| Generalized Linear Model (GLM) | Classification/Regression | None |
| Bagged CART (treebag) | Classification, Regression | None |
| Naive Bayes (nb) | Classification | fL, usekernel, adjust |
| k-Nearest Neighbors (KNN) | Classification, Regression | k |
| C5.0 (C5.0) | Classification | trials, model, winnow |

Reference to Book

[6] M. Kuhn and J. Kjell, *Applied Predictive Modeling*. Springer New York, 2013.
